# NF-κB in the Radiation Response of A549 Non-Small Cell Lung Cancer Cells to X-rays and Carbon Ions under Hypoxia

**DOI:** 10.3390/ijms25084495

**Published:** 2024-04-19

**Authors:** Hasan Nisar, Paulina Mercedes Sanchidrián González, Frederik M. Labonté, Claudia Schmitz, Marie Denise Roggan, Jessica Kronenberg, Bikash Konda, François Chevalier, Christine E. Hellweg

**Affiliations:** 1 Department of Radiation Biology, Institute of Aerospace Medicine, German Aerospace Center (DLR), 51147 Cologne, Germany; hasanisar@pieas.edu.pk (H.N.); pm.sanchidrian@gmail.com (P.M.S.G.); jessica.kronenberg@dlr.de (J.K.); bikash.konda@dlr.de (B.K.); 2 Department of Medical Sciences, Pakistan Institute of Engineering and Applied Sciences (PIEAS), Islamabad 44000, Pakistan; 3 German Center for Neurodegenerative Diseases (DZNE), 53127 Bonn, Germany; 4 Microgravity User Support Center (MUSC), German Aerospace Center (DLR), 51147 Cologne, Germany; 5 UMR6252 CIMAP, CEA-CNRS-ENSICAEN-University of Caen Normandy, 14000 Caen, France; francois.chevalier@ganil.fr

**Keywords:** A549, NF-κB, hypoxia-induced radioresistance, high-LET radiation, ^12^C ions, tumor hypoxia, p65 (RelA) nuclear localization, IL-6/IL-8 secretion

## Abstract

Cellular hypoxia, detectable in up to 80% of non-small cell lung carcinoma (NSCLC) tumors, is a known cause of radioresistance. High linear energy transfer (LET) particle radiation might be effective in the treatment of hypoxic solid tumors, including NSCLC. Cellular hypoxia can activate nuclear factor κB (NF-κB), which can modulate radioresistance by influencing cancer cell survival. The effect of high-LET radiation on NF-κB activation in hypoxic NSCLC cells is unclear. Therefore, we compared the effect of low (X-rays)- and high (^12^C)-LET radiation on NF-κB responsive genes’ upregulation, as well as its target cytokines’ synthesis in normoxic and hypoxic A549 NSCLC cells. The cells were incubated under normoxia (20% O_2_) or hypoxia (1% O_2_) for 48 h, followed by irradiation with 8 Gy X-rays or ^12^C ions, maintaining the oxygen conditions until fixation or lysis. Regulation of NF-κB responsive genes was evaluated by mRNA sequencing. Secretion of NF-κB target cytokines, IL-6 and IL-8, was quantified by ELISA. A greater fold change increase in expression of NF-κB target genes in A549 cells following exposure to ^12^C ions compared to X-rays was observed, regardless of oxygenation status. These genes regulate cell migration, cell cycle, and cell survival. A greater number of NF-κB target genes was activated under hypoxia, regardless of irradiation status. These genes regulate cell migration, survival, proliferation, and inflammation. X-ray exposure under hypoxia additionally upregulated NF-κB target genes modulating immunosurveillance and epithelial-mesenchymal transition (EMT). Increased IL-6 and IL-8 secretion under hypoxia confirmed NF-κB-mediated expression of pro-inflammatory genes. Therefore, radiotherapy, particularly with X-rays, may increase tumor invasiveness in surviving hypoxic A549 cells.

## 1. Introduction

Lung cancer is the second most diagnosed solid malignancy and the leading cause of cancer mortality, accounting for 25% of all cancer deaths annually. About 85% of all lung cancers have an epithelial cell of origin and are classified as non-small cell lung cancer (NSCLC). Almost two-thirds of lung cancer patients receive radiotherapy [1]. However, local control following conventional irradiation techniques is dismal, with a 5-year overall survival of up to 30% [2]. Cellular hypoxia within tumors is a well-established cause of radioresistance and has been demonstrated to be present in up to 80% of NSCLC tumors through multiple positron emission tomography (PET) studies utilizing hypoxia-specific tracers [3,4,5,6,7]. Molecular adaptations to tumor hypoxia have been correlated with treatment resistance in NSCLC in at least three meta-analyses [8,9,10]. Nonetheless, attempts so far to target hypoxia in NSCLC or otherwise have met only limited success [11].

There is a growing interest in improving radiotherapy outcomes in NSCLC using particle therapy, including high linear energy transfer (LET) charged particles to improve survival and local control and reduce side effects [12,13]. This is because, as opposed to photons, which deliver radiation doses diffusely throughout their course, high-LET charged particles, such as carbon (^12^C) ions, deposit the radiation dose densely along a very narrow range, allowing for dose escalation to the tumor, all the while sparing the surrounding normal tissue [14]. High-LET particle radiation has the added advantage of being oxygen-independent in its cytotoxic effect [15], which makes it more effective against hypoxic cancer cells. This has also been demonstrated in vitro in several tumors, including at least one study on NSCLC [16,17]. However, simply ascribing lower tumor resistance against high-LET radiation to its relative physical independence from oxygen for causing cytotoxicity is an oversimplification. In fact, chronic hypoxia is known to induce various cellular adaptations that may enhance radioresistance through modulation of cellular proliferation and survival, as well as tumor invasiveness and metastatic potential [18]. In this regard, the cellular pathways governing hypoxia-induced radioresistance to high-LET radiation such as carbon ions require greater understanding.

Nuclear factor κB (NF-κB) signaling is the primary inflammatory pathway in mammalian cells and is known to be activated by ionizing radiation [19,20,21], as well as cellular hypoxia [22,23,24,25,26]. The NF-κB transcription factor exists in an inactive state in the cytoplasm, sequestered to an inhibitory protein, IκBα. Appropriate stimuli phosphorylate IκBα leading to its degradation, freeing the NF-κB dimer to translocate into the nucleus, thereby activating NF-κB-induced transcription. Ionizing radiation is known to activate NF-κB via the “genotoxic stress-induced pathway” triggered by radiation-induced DNA double-strand breaks (DSBs) [27]. Additionally, cellular stressors such as irradiation and hypoxia can activate NF-κB via the “canonical pathway”, triggered by the release of cytokines such as tumor necrosis factor-α (TNF-α) and interleukin-1 (IL-1) or of damage-associated molecular patterns (DAMPs) [19,24]. Both pathways ultimately converge to translocate activated NF-κB heterodimers from the cytoplasm into the nucleus comprising of the subunit p65 (RelA) bound to the p50 subunit. The p65 subunit contains the main transactivating domain responsible for the function of NF-κB as a transcription factor. Over 200 genes are responsive to the p65:p50 heterodimer, generating a pro-inflammatory response to radiation and hypoxia [28].

Depending upon the cell type, the NF-κB pathway can regulate tumor radioresistance by modulating apoptosis, proliferation, invasion, angiogenesis, and immune surveillance [29,30]. It may be particularly relevant to high-LET irradiation, as NF-κB activation by ionizing radiation is described as dose- and LET-dependent, generally requiring doses above 7 Gy of low-LET radiation but much lower doses of high-LET radiation [31,32]. A LET of 100–300 keV/µm has been reported to have a nine-times higher potential of activating NF-κB in normal human cells compared to low-LET X-rays [19]. With regard to tumor hypoxia, knowledge about the impact of high-LET particle radiation on the NF-κB-signaling pathway is very limited [33]. A tumor-promoting NF-κB-dependent inflammatory response has been reported in breast, pancreatic, rectal, and colon cancer cells [34,35].

However, not much is known about the role of the NF-κB pathway in the response of NSCLC to radiation exposure, particularly after irradiation with high-LET particle radiation and especially in the presence of tumor hypoxia. A greater understanding of the NF-κB-dependent transcriptional response and target protein synthesis in an NSCLC cell line following exposure to clinically relevant high-LET radiation in chronically hypoxic cells may contribute toward the optimization of radiotherapy options in NSCLC. The A549 cell line is a highly used model cell line to study NSCLC. A549 cells are p53 wildtype, carry a Kirsten rat sarcoma viral oncogene homolog (KRAS) missense mutation (KRAS-G12S), and are histologically characterized as adenocarcinoma [36]. KRAS belongs to the significantly mutated oncogenes in NSCLC, as they account for 30% of lung adenocarcinomas in Western countries and for 10–15% of cases in Asia [37,38,39]. As A549 cells carry a relevant KRAS driver mutation and their NF-κB activation is well-characterized [40,41,42,43], we chose this NSCLC cell line for this study, which aimed to compare the effect of high- (carbon ions) and low-LET radiation (X-rays) under hypoxia (1% O_2_) and normoxia (20% O_2_) on the transcriptional response of p65 target genes, as well as p65 target cytokines.

## 2. Results

### 2.1. Expression of NF-κB Target Genes in A549 Cells after Exposure to X-rays or Carbon Ions under Normoxia and Hypoxia

**Figure 1:**
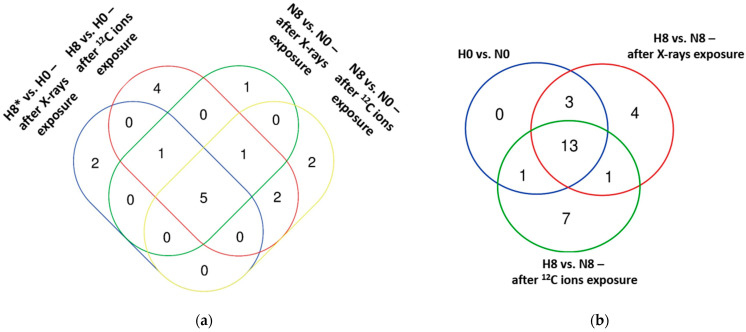
Venn diagrams of differentially upregulated NF-κB target genes in A549 cells after exposure to X-rays or ^12^C ions; (**a**) effect of hypoxia in irradiated and unirradiated cells; (**b**) effect of irradiation in hypoxic and normoxic cells. * H0, A549 cells exposed to 0 Gy X-rays or ^12^C ions under hypoxia; N0, A549 cells exposed to 0 Gy X-rays or ^12^C ions under normoxia; H8, A549 cells exposed to 8 Gy X-rays or ^12^C ions under hypoxia; N8, A549 cells exposed to 8 Gy X-rays or ^12^C ions under normoxia. n = 4.

A search was made for NF-κB target genes listed in ChIP-X enrichment analysis (CHEA) and ENCORE databases (Section 4.3) among the statistically significant differentially upregulated genes observed in our study four hours after irradiation (using X-rays and ^12^C ions) in comparison to unirradiated controls under normoxia and hypoxia in our experiments (Figure 1a). The same was done for differentially upregulated genes observed in irradiated and unirradiated hypoxic cells compared to irradiated and unirradiated normoxic controls, respectively (Figure 1b).

Five NF-κB target genes were upregulated following irradiation, irrespective of oxygenation status or type of radiation (Figure 1a), of which FAS cell surface death receptor (*FAS*) and Cyclin-dependent kinase 1A (*CDKN1A*) showed the highest upregulation in comparison to unirradiated controls. Log_2_ fold change for *FAS* was highest, while that of *CDKN1A* was lowest following carbon ions exposure under hypoxia (Table 1). In comparison to unirradiated controls, four genes were exclusively upregulated following carbon ion exposure under hypoxia. Of these, arachidonate 5-lipoxygenase (*ALOX5*) exhibited the highest log_2_ fold change of 2.49 (Table 1). Two genes were exclusively upregulated following X-ray irradiation under hypoxia (Figure 1a) compared to unirradiated controls, with platelet-activating factor receptor (*PTAFR*) showing the greatest log_2_ fold change of 1.15. In contrast, under normoxia, two genes were exclusively upregulated after carbon ions exposure when compared to corresponding unirradiated controls (Figure 1a). Of these, empty spiracles homeobox 1 (*EMX1*) showed the greatest log_2_ fold change of 3.10 (Table 1); only one gene was exclusively upregulated after X-ray exposure under normoxia (Figure 1a) compared to corresponding unirradiated controls, namely, growth arrest and DNA damage-inducible alpha (*GADD45A*) with a log2 fold change of 1.11 (Table 1). Overall, carbon ion irradiation led to differential upregulation of a greater number of NF-κB target genes compared to X-ray exposure under both normoxia (10 vs. 8, respectively) and hypoxia (13 vs. 8, respectively).

There was an overlap of 13 NF-κB responsive genes that were upregulated by hypoxia, independent of irradiation status (Figure 1b). Seven genes were exclusively upregulated under hypoxia compared to normoxia after exposure to carbon ions (Figure 1b), of which angiotensinogen (*AGT*) and phosphoglycerate kinase 1 (*PGK1*) showed the highest log_2_ fold change increase (2.88 and 1.49, respectively; Table 1).

Similarly, X-ray exposure of hypoxic cells led to four genes being exclusively upregulated (Figure 1b), of which C-C motif chemokine ligand 20 (*CCL20*) and Lipase G (*LIPG*) expressed the highest log_2_ fold change increase (2.05 and 1.47, respectively; Table 1). The only gene that was commonly upregulated under hypoxia following either X-rays or carbon ion exposure (Figure 1b; Table 1) was the CD274 molecule (*CD274*). Overall, hypoxia led to differential upregulation of 17 NF-κB target genes in comparison to normoxia, while irradiation under hypoxia using carbon ions and X-ray exposure upregulated a similar number of genes (22 vs. 21, respectively) compared to irradiation under normoxia.

### 2.2. Secretion of the Cytokines IL-6 and IL-8 by A549 Cells after Exposure to X-rays or Carbon Ions under Normoxia and Hypoxia

Since the genes for both IL-6 (*IL6*) and IL-8 (*CXCL8*) are NF-κB responsive and were found to be upregulated under hypoxia compared to normoxia, both in the absence and presence of radiation exposure using either X-rays or ^12^C ions (Table 1), IL-6 and IL-8 levels were measured by ELISA. To account for differences in the cell number under hypoxia and normoxia, and after irradiation due to different proliferation and cell death, the amount of the cytokine in the supernatants was normalized to one million cells for each sample.

**Figure 2:**
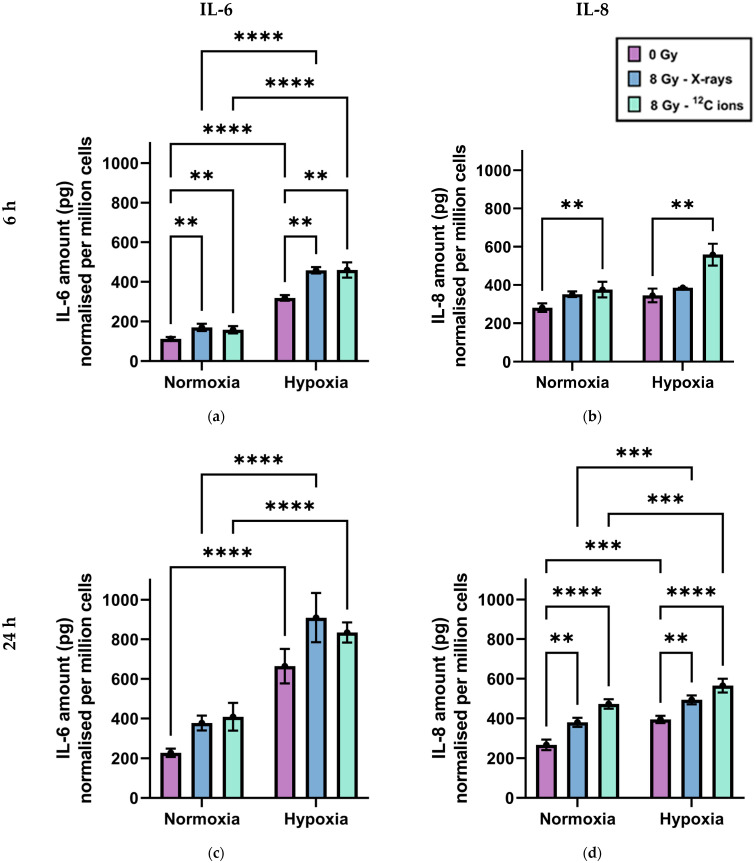
IL-6 and IL-8 content of cell culture supernatants of A549 cells after exposure to X-rays or carbon ions under normoxia and hypoxia; (**a**) IL-6; 6 h (**b**) IL-8; 6 h (**c**) IL-6; 24 h (**d**) IL-8; 24 h. Mean (filled circle) ± SE of cytokine secreted within 6 h or 24 h by one million cells are shown for n = 3–6. Significance was tested using two-way ANOVA and Sidak multiple comparison tests with ** *p* < 0.01, *** *p* < 0.001, **** *p* < 0.0001.

Chronic hypoxia increased IL-6 and IL-8 secretion by A549 cells, independently of irradiation (Figure 2). Twenty-four hours after a medium change in the absence of radiation, both IL-6 (Figure 2a,c) and IL-8 levels (Figure 2b,d) were significantly greater in cell culture media when cells were incubated under hypoxia compared to normoxia. Additionally, IL-6 levels in media were significantly higher under hypoxia compared to normoxia, even 6 h after a medium change in the absence of radiation.

Furthermore, irradiation of A549 cells increased IL-6 and IL-8 secretion, independently of oxygenation status. There was no significant difference in cytokine secretion between X-rays and ^12^C ion exposure. This increase in IL-6 secretion was statistically significant 6 h after irradiation, while that of IL-8 secretion was statistically significant 24 h after irradiation.

## 3. Discussion

### 3.1. Irradiation Upregulates NF-κB Target Genes Influencing Apoptosis, Cell Cycle, and Cell Migration in A549 Cells

Five NF-κB responsive genes were identified as being upregulated following irradiation, irrespective of radiation quality and oxygen conditions (Table 1). FAS cell surface death receptor (*FAS*) positively regulates the extrinsic pathway of apoptosis and its overexpression may therefore reduce the survival of A549 cells [44]; however, *FAS* overexpression has also been linked to cancer stem cell promotion [45]. Cyclin-dependent kinase 1A (*CDKN1A*), and Polo-like kinase 3 (*PLK3*) are cell cycle inhibitors, but *CDKN1A* overexpression has also been associated with increased cell migration and cancer stem cell promotion [46]. KIT Ligand (*KITLG*) and Netrin 1 (*NTN1*) influence cell migration. Their overexpression has been reported previously in several cancers, including NSCLC, and is associated with increased cancer cell proliferation and invasiveness [47,48,49].

Therefore, while X-rays and particle therapy are vital for the effective treatment of many solid malignancies such as NSCLC, their use may increase tumor invasiveness in surviving cells. The issue has gained popularity in the last decade [50] and has been reported more recently in NSCLC A549 cells as well, but not in the context of NF-κB target gene overexpression [51].

### 3.2. High-LET Radiation Enhances NF-κB Target Genes Expression in A549 Cells Compared to Low-LET Radiation

Of the five NF-κB responsive genes that were upregulated after irradiation regardless of oxygenation status and radiation quality, four showed greater fold change following ^12^C ion exposure in comparison to X-rays under hypoxia and three under normoxia (Table 1). Quantitatively stronger NF-κB target gene expression to high-LET radiation has been reported previously in HEK cells [33]. High-LET irradiation of normal tissue has been reported to increase oncogenic transformation in comparison to X-rays [52]; therefore, it would be interesting to investigate whether greater fold change response of the five genes mentioned in Section 4.2 would contribute to a greater potential of cancer progression following ^12^C ion exposure of NSCLC cells compared to irradiation with X-rays.

The qualitative influence of radiation type on NF-κB target gene expression appeared minimal, with only two genes found to be upregulated after ^12^C ion exposure, regardless of oxygenation status, that were not upregulated after X-ray irradiation (Table 1). Both genes appear to be involved in the modulation of an inflammatory response to radiation. Transcription factor EC (*TFEC*) regulates a variety of cytokines capable of cancer stem cell promotion [53,54,55], while the pro-cancer or anti-cancer role of Epstein-Barr virus-induced gene 3 (*EBI3*) is less clearly understood [56].

### 3.3. Chronic Hypoxia Upregulates NF-κB Target Genes Influencing Cell Survival and Cell Migration in A549 Cells

Without irradiation, 17 NF-κB responsive genes were upregulated under chronic hypoxia (1% O_2_) compared to normoxia (Table 1). Seven of these encoded cytokines are interleukin 1A and B (*IL1A* & *IL1B*), interleukin 6 (*IL6*), C-X-C motif chemokine ligand 8 (*CXCL8*), platelet-derived growth factor subunit B (*PDGFB*), tissue factor (*F3*) and CC motif chemokine ligand 28 (*CCL28*), all of which are known to promote tumor growth and metastasis through modulation of cell proliferation, cell migration, immunosuppression, and oncogenic transformation. *IL1A* and *IL1B*, as well as *IL6,* may additionally inhibit apoptosis [57,58]. IL-6 has been described as a potential therapeutic target for NSCLC immunotherapy [59,60]. *CXCL8* overexpression has also been described in NSCLC as a cause of tumor invasiveness [61] and has been highlighted as a potential therapeutic target in different cancers [62]. Interestingly, the products of the above-mentioned genes, IL-1α, IL-1β, and IL-6, may also harm A549 survival, as they have been reported to induce apoptosis and necroptosis in many cell lines [63,64,65,66,67,68].

As an antiapoptotic protein, BCL-2-related protein A1 (BCL2A1) may counter-oppose the pro-apoptotic effects of IL-1α, IL-1β, and IL-6, as well as those of caspase recruitment domain family member 11 (*CARD11*) and potassium channel tetramerization domain containing 11 (*KCTD11*) on induction of apoptosis. *KCTD11* overexpression in NSCLC has recently been reported to inhibit cancer progression [69].

Two metalloproteinases, ADAM metallopeptidase domain 19 (*ADAM19*) and matrix metallopeptidase 9 (*MMP9*), upregulated under hypoxia, are also associated with cancer progression, mainly through modulation of cell adhesion and migration in several cancers, including NSCLC [70,71,72,73]. Inhibin subunit beta A (*INHBA*) and SRY-box transcription factor 9 (*SOX9*) are known to influence cell differentiation and have been reported to increase tumor aggressiveness in NSCLC through induction of epithelial-mesenchymal transition (EMT) [74,75,76,77].

JUNB proto-oncogene (*JUNB*) and eosinophil granule ontogeny transcript (*EGOT*) are understood to mainly inhibit cell proliferation and survival. JUNB, as a component of activator protein-1 (AP-1), inhibits the cell cycle and induces senescence [78,79], thereby acting as a tumor suppressor in a variety of cancers. However, its overexpression was found to cause tumor progression in NSCLC [80]. *EGOT* encodes for a long noncoding RNA that has been reported to inhibit cell migration and proliferation in breast and renal cell carcinoma [81,82], but its role in NSCLC is unclear [83].

In summary, except for *KCTD11*, all other NF-κB target genes upregulated in A549 cells due to chronic hypoxia promote cancer progression. None of these 17 genes, except *IL1A,* was found to be regulated by irradiation when compared with their corresponding unirradiated controls, regardless of oxygen concentration.

### 3.4. NF-κB Target Genes’ Activation Signature Following Irradiation May Promote Cancer Progression under Hypoxia, Especially after X-ray Exposure

Following irradiation of hypoxic cells, the NF-κB target genes’ activation signature remained similar to the one observed in hypoxic cells in the absence of irradiation. In comparison to irradiated normoxic controls, of the 17 genes upregulated because of chronic hypoxia (Section 3.3), 13 were found to be regulated following ^12^C ion exposure and 16 following X-ray exposure.

However, irradiation under hypoxia did upregulate the NF-κB responsive gene CD274 antigen (*CD274*) in comparison to irradiated normoxic controls. CD274 antigen, also called programmed death ligand 1 (PD-L1), is crucial for the escape of tumor cells from immunosurveillance [84]. Its upregulation was greater following X-ray exposure than ^12^C ion exposure, indicating a potential benefit of using ^12^C ions over X-rays in treating hypoxic NSCLC. Cellular hypoxia is being increasingly reported to promote an immunosuppressive tumor microenvironment that promotes cancer propagation [85]. PD-L1 overexpression in NSCLC is well-characterized [86,87] and is currently being targeted clinically, with and without radiotherapy [88,89,90]. X-ray exposure of hypoxic A549 cells also upregulated the expression of an EMT transcription factor gene, specifically snail family transcriptional repressor 1 (*SNAI1*), which was recently reported to be associated with tumor progression and aggressiveness in NSCLC [51,91,92]. This may indicate another justification in favor of ^12^C ions treatment over X-rays in targeting hypoxic NSCLC. X-ray exposure under hypoxia compared to that under normoxia also upregulated platelet-activating factor receptor (*PTAFR*), which has been reported to enhance cancer progression in certain tumors [93].

On the other hand, ^12^C ion irradiation of hypoxic cells also led to the upregulation of NF-κB target genes promoting cancer progression in comparison to irradiated normoxic controls. These included phosphoglycerate kinase 1 (*PGK1*) and arachidonate 5-lipoxygenase (*ALOX5*). PGK1 has been reported to promote cancer cell proliferation and migration in NSCLC through the downstream ERK/MCM4 pathway [94]. *ALOX5* is generally downregulated in NSCLC [95], and its overexpression has been reported to be associated with tumor progression in gliomas [96].

Recently, we correlated pro-EMT gene expression signature in hypoxic A549 cells following X-ray exposure in comparison to carbon ion irradiation with greater radioresistance to X-rays in this cell line [97]. To establish whether X-rays exposure may lead to greater cell survival in NSCLC cells than ^12^C ions irradiation via EMT enhancement and even immune escape requires functional (e.g., migration) tests and animal experiments.

### 3.5. Chronic Hypoxia Results in an Inflammatory Response in A549 Cells Distinct from Normoxia, Irrespective of Irradiation Status

IL-6 and IL-8 cytokine secretion by A549 cells under the influence of different oxygen concentrations and radiation qualities was determined to confirm that upregulation of NF-κB target genes manifested into relevant protein synthesis. These cytokines were selected because their secretion is influenced by NF-κB activation and they were linked to cancer cell propagation and survival [24,98,99,100]. Furthermore, their secretion in A549 cells is well-characterized and their serum levels can be used as biomarkers of progression in lung cancer patients [101,102,103]. Recently, many studies tie the release of pro-inflammatory cytokines such as IL-6 and IL-8 with the activation of the cGAS-STING pathway in response to DNA double-strand breaks such as those induced by ionizing radiation [104,105,106,107]. However, our RNA sequencing data showed no differential upregulation of genes encoding key proteins constituting the type 1 interferon response, such as interferons α or β. Furthermore, most studies report IL-6 and IL-8 secretion in response to cGAS-STING pathway activation only as a secondary effect of NF-κB activation through the action of TANK-binding kinase 1 (TBK1) [108,109,110].

Importantly, in our study, secretion of both IL-6 and IL-8 was found to be increased under the influence of chronic hypoxia, as well as that of irradiation, regardless of radiation quality (Figure 2). This aligned perfectly with NF-κB target gene expression data for *IL6* and *CXCL8* genes and may be extrapolated to assume similar changes in cytokine levels because of other upregulated NF-κB responsive genes encoding pro-inflammatory proteins.

The secretion was greatest under the combined effect of both hypoxia and irradiation 6 h after irradiation in the case of IL-6, and 24 h after irradiation in the case of IL-8. Since both IL-6 and IL-8 were shown to positively impact tumor propagation by enhancing immune evasion, angiogenesis, and metastatic potential [60,61,101,111], our findings indicate that both low- and high-LET radiation can augment the tumor-promoting attributes of chronic hypoxia. While cytokine-mediated inflammation is established as an important component of cellular responses to both irradiation and cellular hypoxia in normal and tumor cells [112,113,114,115,116], there was no direct comparison of NF-κB-induced cytokine secretion by cancer cells under normoxia and hypoxia following exposure to high- and low-LET radiation to the best of our knowledge up to now. In a holistic view of the tumor response to the different radiation qualities, the impact on survival has to be considered in addition to radio-induced inflammation, which is higher for carbon ions compared to X-rays, with relative biological effectiveness (RBE) above 2.3 in normoxic and hypoxic A549 cells, as we recently described [97]. Such a higher killing efficiency of carbon ions could counteract the tumor-supporting effects of the hypoxic milieu.

## 4. Materials and Methods

### 4.1. Cell Line and Cultivation

A549 cells (human, male, lung adenocarcinoma, KRAS mutated, p53 wildtype [117]) were purchased from LGC Genomics (Berlin, Germany) and cultured in 25 cm^2^ or 80 cm^2^ cell culture flasks (Labsolute, Th. Geyer GmbH, Renningen, Germany) at a density of 5000 cells/cm^2^, using Alpha-Minimally Essential Medium (α-MEM; PAN Biotech, Aidenbach, Germany) containing 10% (*v*/*v*) dialyzed Fetal Bovine Serum (FBS; PAN Biotech), 2% (*v*/*v*) sterile glucose solution (0.94 mol/L), 1% (*v*/*v*) Penicillin (10,000 U/mL)/Streptomycin (10 mg/mL) (PAN Biotech), 1% (*v*/*v*) Neomycin/Bacitracin (Biochrom AG, Berlin, Germany), and 1% (*v*/*v*) Amphotericin (250 µg/mL) (PAN Biotech). The cells were regularly tested for mycoplasma contamination by polymerase chain reaction of supernatants at the Leibniz-Institut DSMZ- Deutsche Sammlung von Mikroorganismen und Zellkulturen GmbH (Braunschweig, Germany) and were mycoplasma-free.

The cells were incubated at 37 °C and saturated humidity, either under normoxia (20% O_2_) in a CO_2_ incubator (5% CO_2_; Heraeus HERAcell 150, Thermo Fisher Scientific, Karlsruhe, Germany) or under hypoxia (1% O_2_) in an InvivO_2_ 400 hypoxia workstation (Baker Ruskinn, South Wales, UK) flushed with 5% CO_2_, 1% O_2_, and 94% N_2_. The incubation time in culture under normoxia or hypoxia before irradiation was 48 h to allow cells to enter the exponential growth phase. Medium change, fixation, or lysis of hypoxic cells were performed in the hypoxia workstation. Medium and reagents used for the purpose were degassed by warming them to 25 °C in the Sonorex Digiplus ultrasonic water bath (Bandelin, Berlin, Germany) for 40 min, followed by placing them in the hypoxia workstation for another 40 min with loosened bottle caps before use.

### 4.2. Irradiation

After 48 h of incubation, A549 cells were irradiated with either X-rays or ^12^C ions. The caps of the culture flasks were tightened before transferring them for irradiation. The flasks housing hypoxic cells were shifted for irradiation in air-tight boxes before exporting them out of the hypoxia workstation. They were only taken out from the air-tight boxes for the brief minutes of actual irradiation, following which they were returned to the air-tight boxes for their transport back. Several oxygen readings were taken before actual experiments using the Seven2go dissolved oxygen meter S9 (Mettler Toledo, Giessen, Germany) to ensure that this method did not lead to a significant change in oxygen concentration within the medium of the flasks housing the hypoxic cells.

X-ray exposure (LET: 0.3–3.0 KeV/µm) was performed at the Institute of Aerospace Medicine, DLR, Germany, in an RS 225 X-ray chamber (X-strahl, Ratingen, Germany) at a stable dose rate of 1.0 Gy/min, which was ensured by keeping the distance of the sample from the X-ray source to 450 mm. Low-energy X-rays were eliminated using a copper (Cu) filter with a thickness of 0.5 mm. Cells were irradiated either in cell culture flasks (25 cm^2^ or 80 cm^2^) or in cell culture dishes (∅ 3 cm or 6 cm), depending on the specific experiments performed. The dose and dose rate were monitored using the UNIDOS^webline^ dosimeter with the ionization chamber TM30013 (PTW, Freiburg, Germany).

Carbon ion exposure was carried out at the heavy ion accelerator “Grand Accélérateur National de Ions Lourds” (GANIL) in Caen, France, at a dose rate of 1 Gy/min. During carbon ion exposure, cells were placed in the plateau region of the Bragg curve, resulting in constant LET over the thickness of the cells. To attain a LET (in water) relevant for clinical settings (~75 keV/µm), the carbon ion beam energy was reduced from 95 MeV/n to 35 MeV/n by placing a polymethyl methacrylate (PMMA, thickness 16.9 mm) energy degrader in the beam. The energy was further reduced by the polystyrene bottom of the cell culture flask, resulting in an energy of 25.7 MeV/n and a calculated LET in water of 73 keV/µm. The remaining range of the ions in water was 2550 µm, indicating that the cells were exposed in the plateau region of the Bragg curve. The fluence for heavy ions (P/cm^2^) was used to calculate radiation dose (Gy) [97].

Since the cell culture flasks had to be kept upright during carbon ion exposure due to the horizontal beam setup, the flasks were filled to the neck with a culture medium to prevent desiccation of the cells during exposure.

After irradiation, cells underwent a medium change and were then incubated further, either at 20% O_2_ (normoxia) or 1% O_2_ (hypoxia), for variable periods, depending on the specific experiments.

### 4.3. Gene Expression Analysis

To determine the global transcription profile of cells irradiated under normoxia and hypoxia with 8 Gy of X-rays or ^12^C ions, the culture medium was removed 4 h after irradiation, and cells were lysed using RLT buffer (Qiagen, Hilden, Germany) containing β-mercaptoethanol (1:100, Sigma Aldrich, St. Louis, MO, USA). RNA was isolated with the RNeasy Mini Kit (Qiagen). RNA concentration and integrity were determined using the RNA 6000 Nano Assay (Agilent Technologies, Böblingen, Germany) in the Bioanalyzer (Agilent Technologies). There was 3 µg total RNA per sample (4 biological replicates per condition), with RNA Integrity Numbers (RIN) above 9.0 being sent on dry ice to GENEWIZ (Leipzig, Germany) for mRNA sequencing in the same run after Poly(A) selection using the Illumina NovaSeq6000 platform (configuration: 2 × 150 bp, 350 M read pairs). GENEWIZ mapped the reads onto the *Homo sapiens* GRCh38 reference genome and calculated unique gene hit counts falling within exon regions. Then, the DESeq2 package in R [118] was utilized for differential gene expression analysis. Gene Set Enrichment Analysis (GSEA) was performed using the expression data [119]. Genes with an adjusted *p*-value < 0.05 and absolute log_2_ fold change > 1 were considered as differentially expressed genes for each group comparison. We searched for NF-κB responsive genes among the DEGs using CHEA and ENCORE databases as references, with the help of the online tool Harmonizome [120].

### 4.4. Quantification of Cytokines

Cytokine secretion by A549 cells under normoxia and hypoxia with and without irradiation with X-rays and ^12^C ions was assessed by ELISA. Human IL-6 and IL-8 uncoated ELISA Kits (Invitrogen, Thermo Fisher Scientific, Karlsruhe, Germany) were used for IL-6 and IL-8 detection in sample supernatants.

At the defined time points (6 and 24 h), the supernatants (3 mL) were collected in Eppendorf tubes and stored at −80 °C until subsequent handling. The cells on the other hand were trypsinized and counted with the LUNA automated cell counter for normalization of cytokine production to the cell number.

Ninety-six-well plates (Corning^TM^ Costar^TM^ 9018 ELISA plate, Kaiserslautern, Germany) were coated with the primary capture antibodies (100 μL) provided with the kits diluted 1:250 in PBS and the plates were incubated at 4 °C overnight. Afterward, the wells were blocked for nonspecific antibody binding using diluent (200 μL), which was also provided with the kits, diluted (1:5) in deionized water. The plates were again incubated at 4 °C overnight after loading the wells with the samples (100 µL), as well as several different known dilutions of the provided standard. On the next day, the detection antibody (100 µL per well) provided with the kits was diluted (1:250) in PBS and added to the wells. The well plates were then incubated for 1 h at room temperature. This was followed by incubation at room temperature for 30 min after the addition of Streptavidin-HRP (100 µL), diluted 1:100 for IL-6, or by the addition of Avidin-HRP (100 µL), diluted 1:250 for IL-8. Finally, the plates were incubated for 15 min at room temperature after the addition of TMB substrate (100 µL) to the wells, after which the enzyme reaction was stopped by adding 2 N H_2_SO_4_ (100 µL) to the wells. All incubation steps were carried out on a shaker with 3 to 5 washings in between with wash buffer (PBS with 0.05% Tween).

Plates were read in a Multiskan FC microplate reader (Thermo Fischer Scientific, Waltham, MA, USA) at 450 and 570 nm at 21 °C. Wavelength subtraction (Absorbance A (450 nm)–B (570 nm)) was done via the SkanIt Software (ver. 6.1.1.7, Thermo Fischer Scientific) and displayed in an exportable table for further statistical analyses using Microsoft Excel to plot the standard curves and use them to derive actual cytokine concentrations from the absorbance values.

### 4.5. Statistical Analysis

Three independent biological experiments with three technical replicates for each experimental condition were conducted for the experiments described in Section 4.2, Section 4.3 and Section 4.4. Arithmetical means, standard deviations, and standard errors of means (SE) were calculated using Microsoft^®^ Excel^®^ 2019 MSO software version 16.0.10409.20028 64-Bit (Microsoft corporation, Redmond, WA, USA). Graphs were plotted and tests of significance were performed using GraphPad Prism 9 (Dotmatics, Boston, MA, USA). Significance was tested using one-way analysis of variance (ANOVA) test to evaluate cell metabolism studies and multiple two-way unpaired *t*-tests were used to evaluate cell cycle distribution data and doubling times. Growth curves were plotted using Sigma Plot 15 (Systat Software Inc., Palo Alto, CA, USA). For the RNA sequencing, 4 independent biological experiments were conducted, and a batch analysis of the results was performed.

## 5. Conclusions

Irradiation of hypoxic A549 NSCLC cells using both low- and high-LET radiation leads to an increase in NF-κB-mediated mRNA transcription and cytokine release that may increase cancer cell survival and propagation. The transcriptional response of NF-κB target genes upregulated by both X-rays and ^12^C ions is greater following ^12^C ion exposure. However, X-ray irradiation upregulates a greater number of NF-κB responsive genes involved in inducing oncogenic transformation and enhancing cancer cell survival. Whether this contributes in a major way toward hypoxia-induced radioresistance to X-rays requires further investigation using several NF-κB reporters and knock-down of NF-κB target genes in several NSCLC cell lines to perform functional assays especially related to cell survival and cell migration. Such experiments may be of crucial importance to be able to fully exploit the use of high-LET particle therapy in cancer treatment and to overcome tumor hypoxia-induced radioresistance.

## Figures and Tables

**Table 1:** Differentially regulated NF-κB responsive genes in A549 cells after exposure to X-rays or ^12^C ions.

Regulated DEGs (Gene Name Abbreviation)	Gene Expression Based on *p*-Adjusted log_2_ Fold Changes						
	Irradiated vs. Unirradiated				Hypoxic vs. Normoxic		
	X-rays		^12^C Ions		Controls (H0 vs. N0)	X-rays (H8 vs. N8)	^12^C Ions (H8 vs. N8)
	Normoxia (N8 ^a^ vs. N0)	Hypoxia (H8 vs. H0)	Normoxia (N8 vs. N0)	Hypoxia (H8 vs. H0)			
*FAS*	**2.06**	**2.75**	**2.31**	**2.85**	−0.71	−0.02	−0.17
*NTN1*	**1.36**	**1.93**	**2.19**	**2.29**	−0.03	0.54	0.07
*CDKN1A*	**2.63**	**2.62**	**2.61**	**2.38**	−0.19	−0.21	−0.42
*KITLG*	**1.01**	**1.38**	**1.36**	**1.46**	−1.10	−0.73	−1.00
*PLK3*	**1.28**	**1.14**	**1.13**	**1.16**	−0.26	−0.40	−0.23
*IL1A*	**1.37**	**1.54**	0.43	**1.26**	**2.63**	**2.81**	**3.46**
*EBI3*	0.60	0.81	**1.04**	**1.45**	−0.29	−0.08	0.12
*TFEC*	2.49	N/A ^b^	**2.95**	**2.87**	N/A	N/A	N/A
*REV3L*	0.78	**1.08**	0.79	0.89	−0.43	−0.13	−0.33
*PTAFR*	0.83	**1.15**	0.72	0.97	−0.55	−0.23	−0.29
*GAD1*	0.70	0.76	0.69	**1.32**	−0.63	−0.57	0.00
*BAX*	0.58	0.83	0.74	**1.01**	−0.27	−0.03	0.00
*MX1*	0.22	0.18	0.12	**1.16**	−0.31	−0.35	0.73
*ALOX5*	1.16	1.35	1.43	**2.49**	0.48	0.68	**1.54**
*GADD45A*	**1.11**	0.95	0.77	0.55	0.18	0.02	−0.04
*CCR7*	0.76	0.49	**1.02**	0.62	0.00	−0.27	−0.40
*EMX1*	1.33	0.48	**3.10**	2.55	−0.71	−1.56	N/A
*KCTD11*	0.26	−0.02	0.16	0.34	**1.96**	**1.67**	**2.14**
*ADAM19*	−0.19	−0.04	−0.15	−0.02	**1.46**	**1.60**	**1.58**
*INHBA*	0.15	0.28	0.05	0.19	**1.41**	**1.55**	**1.55**
*MMP9*	0.26	0.81	−0.45	−0.75	**1.87**	**2.43**	**1.58**
*PDGFB*	−0.17	0.15	−0.21	−0.08	**1.63**	**1.95**	**1.75**
*F3*	−0.05	−0.02	−0.62	0.03	**2.00**	**2.02**	**2.65**
*CXCL8*	−0.15	0.03	0.01	0.10	**1.31**	**1.49**	**1.41**
*SERPINE1*	0.37	0.57	0.09	0.36	**1.64**	**1.84**	**1.91**
*IL6*	0.37	0.32	0.65	0.70	**1.23**	**1.18**	**1.28**
*CARD11*	0.00	0.14	−0.17	0.20	**1.05**	**1.18**	**1.42**
*BCL2A1*	0.54	0.58	0.19	−0.17	**1.54**	**1.59**	**1.18**
*JUNB*	−0.18	−0.03	−0.10	0.09	**1.21**	**1.36**	**1.40**
*EGOT*	0.73	1.03	−1.68	0.65	**1.94**	**2.24**	N/A
*IL1B*	0.56	1.33	1.94	0.19	**1.80**	**2.58**	0.05
*SOX9*	−0.57	−0.11	0.12	−0.11	**1.01**	**1.47**	0.79
*CCL28*	0.83	−0.19	−0.34	0.26	**1.49**	0.47	**2.09**
*CD274*	−0.26	−0.06	−0.26	−0.11	0.91	**1.11**	**1.05**
*TRAF1*	0.16	0.51	0.19	−0.21	0.98	**1.32**	0.58
*SNAI1*	−0.68	0.25	−0.52	−0.21	0.32	**1.24**	0.63
*CCL20*	−1.45	−0.02	0.63	−0.10	0.62	**2.05**	−0.11
*LIPG*	−0.28	0.66	−0.09	0.21	0.53	**1.47**	0.83
*ENO2*	0.06	−0.20	−0.29	−0.02	0.82	0.56	**1.08**
*CCL2*	0.16	0.27	−0.02	0.25	0.89	1.00	**1.17**
*PGK1*	0.24	0.03	−0.49	0.08	0.92	0.70	**1.49**
*AGT*	0.39	−0.38	−1.30	1.56	0.02	−0.75	**2.88**
*ICAM1*	0.19	0.45	−0.23	0.33	0.52	0.78	**1.09**
*GADD45B*	0.07	0.01	−0.03	0.16	0.99	0.93	**1.19**

^a^ N8, A549 cells exposed to 8 Gy X-rays or ^12^C ions under normoxia; N0, A549 cells exposed to 0 Gy X-rays or ^12^C ions under normoxia; H8, A549 cells exposed to 8 Gy X-rays or ^12^C ions under hypoxia; H0, A549 cells exposed to 0 Gy X-rays or ^12^C ions under hypoxia; ^b^ N/A, used if log_2_ Fold Change values were missing in RNA sequencing data. Statistically significant DEGs are given in bold. Cells were preincubated for 48 h before irradiation and RNA was extracted 4 h after irradiation.

## Data Availability

Research data are stored in an institutional repository and will be shared upon request to the corresponding author.
